# Bionic scaffolds with integrated structural components based on low‐temperature deposition manufacturing 3D printing technology for the treatment of meniscus defects

**DOI:** 10.1002/btm2.70022

**Published:** 2025-04-25

**Authors:** Shi Shen, Yujie Li, Mingxue Chen, Weimin Guo, Shuang Gao, Zengzeng Zhang, Naiqiang Zhuo, Shuyun Liu, Quanyi Guo

**Affiliations:** ^1^ Chinese PLA General Hospital, Beijing Key Lab of Regenerative Medicine in Orthopedics, Key Laboratory of Musculoskeletal Trauma & War Injuries PLA Institute of Orthopedics Beijing People's Republic of China; ^2^ Department of Orthopedics, The Affiliated Hospital Southwest Medical University Luzhou People's Republic of China

**Keywords:** 3D printing, integration, low‐temperature deposition manufacturing, meniscus tissue engineering, PCL/COL I

## Abstract

Tissue engineering provides a promising avenue for treating meniscus defects. In this study, a novel polycaprolactone (PCL)/collagen type I (COL I) meniscus scaffold was fabricated using low temperature deposition manufacturing (LDM) 3D printing technology. The scaffold had a ring and radial fiber structure, and its composition and structure were double bionic of the natural meniscus. In vitro experiments showed that the scaffold had good biological properties, which could promote the proliferation of meniscus fibrochondrocytes (MFCs) and increase the secretion of collagen and glycosaminoglycan. Moreover, the scaffold had excellent mechanical properties and could withstand various stress loads from the femur and tibia. The integrity of the scaffold structure was maintained to provide sufficient time and space for tissue regeneration. The PCL/ COL I scaffold has shown good therapeutic effect in a rabbit meniscus defect model and promotes meniscus regeneration. The results of experiments in rabbits suggest that the scaffold may recruit stem cells and differentiate into fibrochondrocytes in the knee joint, which needs to be verified by further experiments. This study introduces a method of fabricating a new structural composition double bionic meniscus scaffold by LDM technology and verifies its ability to promote cell proliferation, increase the secretion of the extracellular matrix of fibrocartilage, and regulate the microenvironment of cell growth. In addition, this scaffold has achieved good results in repairing meniscus defects in small animal models. Our findings strongly indicate that the PCL/COL I biomimetic meniscus scaffold prepared using 3D‐LDM technology holds great promise for repairing and regenerating damaged menisci.

**Translational Impact Statement**

The PCL/COL I biomimetic meniscus scaffold represents a significant advance in orthopedic tissue engineering, addressing both biological and mechanical demands of meniscus repair. Its translational impact lies in bridging laboratory innovation to clinical practice, offering a sustainable, patient‐tailored solution for improving joint health outcomes. In conclusion, the PCL/COL I biomimetic meniscus scaffold prepared using 3D‐LDM technology holds great promise for repairing and regenerating damaged menisci.

## 1 INTRODUCTION

The meniscus is one of the important organs to ensure the normal function of the knee joint. Its main functions include absorbing shock, transferring stress loads, lubricating cartilage, and stabilizing the knee joint.^1^, ^2^ Due to the special anatomical structure of the meniscus, only 10%–25% of its surrounding area has blood supply.^3^, ^4^, ^5^ Therefore, once the meniscus tissue is damaged, its self‐repair ability is very limited. Clinically, suturing can be used as a surgical method to promote meniscus healing after minor injury or tear in the blood supply area of the meniscus.^6^, ^7^ For medial meniscus injury in the non‐blood supply area, only palliative surgery such as partial or complete meniscectomy can be used. Moreover, it only provides short‐term symptom relief and functional recovery, ultimately leading to a significantly increased risk of developing osteoarthritis.^8^, ^9^, ^10^, ^11^ Meniscus allograft transplantation is a viable treatment option, but its broad clinical use is limited by problems such as limited sources, difficulties in matching morphology and size, and the possibility of immune rejection and disease transmission.^12^, ^13^, ^14^ Therefore, regenerative repair of meniscus injury remains a challenging scientific problem.

Over the past two decades, tremendous progress has been made in tissue engineering technology, and numerous studies have shown that biomimetic tissue engineering exhibits great potential in the repair and regeneration of various human tissue and organ diseases. At present, it is generally believed that meniscus tissue engineering technology is expected to solve the challenging problem of meniscus regeneration and repair. The three key elements of tissue engineering include scaffolds, seed cells, and cellular active factors.^15^, ^16^, ^17^ However, scaffold materials remain a major limiting factor for the development of meniscus tissue engineering. Synthetic polymer materials have attracted much attention due to their good mechanical properties, strong ductility, biodegradability, and abundant sources. However, the major disadvantages include poor biocompatibility, low cellular affinity, and potential toxicity associated with the degradation products of scaffold materials.^18^ Autologous materials face challenges such as limited sources, donor site pain, and potential complications. Allogeneic materials have risks such as disease transmission and immune rejection. In addition, materials from different tissue sources show significant biomechanical differences, and their limited plasticity makes it difficult to match the unique morphological structure of the meniscus.^19^, ^20^ Biological scaffolds have the advantages of good biocompatibility, high cell affinity, and high degradability, which is conducive to the adhesion and proliferation of seed cells in the later stage. However, it also has disadvantages such as poor mechanical properties and a fast degradation rate.^21^, ^22^ In conclusion, it is difficult for a single material to meet the requirements of meniscus tissue engineering scaffold at the same time.

Therefore, the development of a composite bionic meniscus scaffold with excellent biocompatibility and mechanical properties will bring new hope for the treatment of meniscus injuries. Polycaprolactone (PCL) is a multifunctional biomaterial that has been approved by the FDA for clinical use. It possesses excellent compatibility with organic polymers and biodegradability. It is currently widely used in applications such as drug delivery devices, sutures, and adhesion barriers.^23^ By adjusting the concentration of PCL in composite materials, it is possible to achieve mechanical properties comparable to those of a normal meniscus while maintaining an appropriate degradation rate. Zhang ZZ et al. and Gokhan Bahcecioglu et al. successfully prepared a meniscus scaffold with good physical properties using PCL as the raw material.^24^, ^25^

But one major drawback of PCL is that it lacks biofunctional groups necessary for cells to adhere. Hebin Ma et al. utilized Fibrin (FIB) and PCL as raw materials to create a PCL/FIB composite scaffold, which not only improved the poor biocompatibility of the PCL scaffold but also provided a favorable environment for the proliferation of chondrocytes.^26^ The outer periphery of the meniscus is mainly fibrous and contains a high amount of type I collagen (COL 1) (70%–80% of the total collagen), and COL 1 is also a major component of the extracellular matrix (ECM), forming the cellular microenvironment of meniscus fibrochondrocytes (MFCs) while maintaining the tensile properties of the meniscus.^27^, ^28^ COL I exhibits excellent cell adhesion capabilities and high plasticity, but its elastic modulus is relatively low, making it difficult to withstand external loads. Therefore, using PCL and COL I as the fundamental materials for meniscus scaffolds can compensate for the mechanical deficiencies of COL I while enhancing the cell adhesion properties of PCL.

3D printing (3DP) is a promising technology to precisely fabricate customized scaffolds with complex architectures. Compared to traditional 3D‐printing technologies such as stereo lithography apparatus (SLA), selective laser sintering (SLS), 3DP, and fused deposition modeling (FDM), LDM is a technique that utilizes a controlled cooling chamber to maintain low temperatures during the deposition of composite/mixed solutions.^29^ The advantage of LDM technology lies in its ability to be used for the printing of biomaterials, to achieve high‐precision composite slurry deposition, and to maintain the activity of biological factors or cells. Previous studies have used LDM technology to prepare poly (lactic‐co‐glycolic acid) (PLGA) cartilage tissue engineering scaffolds.^30^ In this experiment, the PCL/COL I integrated meniscus scaffold was prepared by LDM‐3DP technology. The structure and composition of the PCL/ COL I integrated meniscus scaffold were double bionic of the natural meniscus. Subsequently, the biomechanical properties, biocompatibility, and the effect of regenerative repair of meniscus defects in rabbits were evaluated.

## 2 MATERIALS AND METHODS

### 2.1 Preparation of PCL‐ COL I scaffolds

Preparation of materials for PCL‐COL I scaffolds, preparation of pre‐printed 3D models, and fabrication of biomimetic PCL‐COL I scaffolds.

#### 2.1.1 Preparation of meniscus scaffold materials

Weigh 1.2, 1.6, 1.8, and 2 g of PCL particles, and add them to solutions containing 0.8, 0.4, 0.2, and 0 g of COL I, respectively, in a mixture of hexafluoroisopropanol (HFIP) and 1,4‐dioxane (with a ratio of 1:4–0:10). This will prepare five different mass ratios of PCL/COL I solutions (Table 1).

**TABLE 1:** Composition of PCL/COL I solutions with different mass ratios.

Group	PCL (g)	COL I (g)	Total mass (g)	HFIP (ml)	1, 4‐dioxane (ml)	Ratio
1	1.2	0.8	2	2	8	60%PCL/40%COL I
2	1.6	0.4	2	1	9	80%PCL/20%COL I
3	1.8	0.2	2	0.5	9.5	90%PCL/10%COL I
4	2	0	2	0	10	100%PCL

#### 2.1.2 3D‐printing preparation of bionic cell‐free PCL‐COL I meniscus scaffold

**FIGURE 1:**
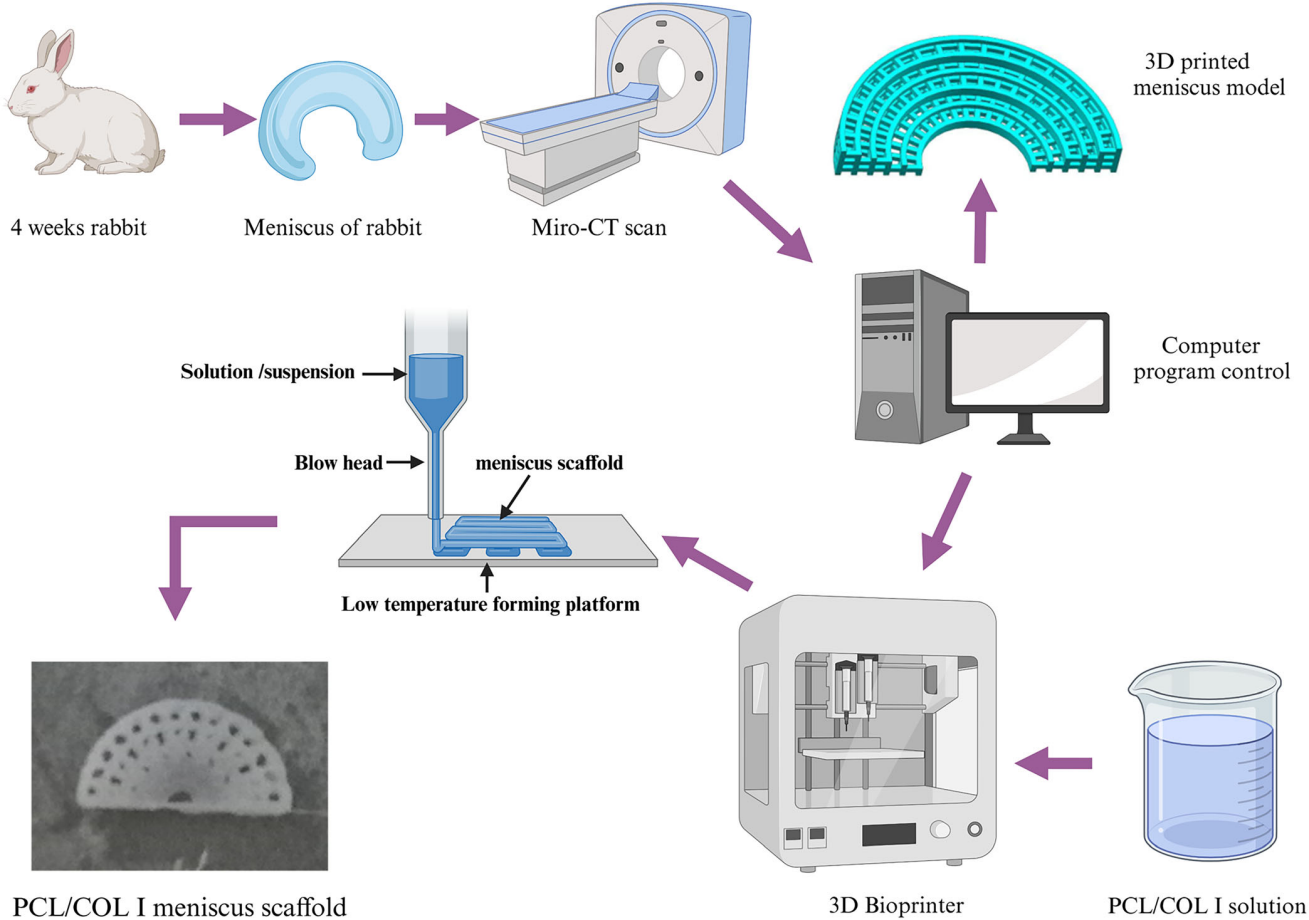
Preparation process of PCL/COL I meniscus scaffold.

Remove the medial meniscus of 1 adult New Zealand white rabbit and perform Micro‐CT scanning. Import the scanned data into the software UG and process and optimize it into a ring‐shaped radial wedge‐shaped meniscus scaffold model. Subsequently, import the model data into a 3D bioprinter in STL format to prepare the scaffold (Figure 1).

### 2.2 Physical property testing of PCL/COL I meniscus scaffold

The PCL/COL I scaffold's macro and micro structures, components, biomechanical properties, and hydrophilicity were detected by physical experiments.

#### 2.2.1 Observation of scaffold morphology and microstructure

Observe the general morphology of the scaffold, then freeze‐dry it. Attach the scaffold to an aluminum circular stage using conductive adhesive. After spraying gold on the scaffold surface, observe its microstructure using a Hitachi S‐4800 scanning electron microscope.

#### 2.2.2 FTIR analysis

After freeze‐drying the PCL/COL type I collagen solution for 24 h, a sample identical to the five scaffold components was obtained. The sample was cut into 10 mm × 10 mm square slices, and pure COL I powder was used as a control. FTIR analysis was performed in reflection mode.

#### 2.2.3 Mechanical property testing

Mechanical testing was conducted using a BOSE 5100 biomechanical testing machine. 1. Prepare cylindrical scaffold samples measuring 10 mm × 10 mm × 5 mm, and conduct compressive mechanical testing after soaking them in PBS buffer solution. 2. Prepare rectangular scaffold samples measuring 16 mm × 10 mm × 2 mm, and conduct tensile mechanical testing after soaking them in PBS buffer solution.

#### 2.2.4 Surface contact angle measurement

PCL/COL type I collagen solution was freeze‐dried for 24 h, and then a 10 × 10 mm square sample was prepared. Subsequently, the sample was placed on a glass slide, and deionized water was dropped onto the sample surface using a microsyringe equipped with a 27G blunt needle. The drop analysis system captured the image of the falling deionized water droplets. The surface contact angle was calculated based on the shape of the water droplets.

### 2.3 Biocompatibility evaluation

Evaluate the scaffold's cytotoxicity, cell viability, cell adhesion ability, and its ability to maintain cell phenotype and growth microenvironment through in vitro cell experiments.

#### 2.3.1 CCK‐8 cytotoxicity assay

Harvest the meniscus from the knee joint of New Zealand white rabbits (4 weeks old), remove the lateral synovial tissue and the peripheral 1/3 area of the meniscus, isolate the rabbit MFCs, and culture them to P3 generation cells for future use. Immerse the scaffold sample in DMEM/F12 medium, incubate it at (37 ± 1)°C for 72 h, centrifuge, collect the supernatant, and then add 10% (v/v) FBS and 1% (v/v) penicillin–streptomycin double antibiotic. Inoculate MFCs into 96‐well plates, adding 100 μL of extracting solution per well. Use cells cultured in normal medium as negative controls and cells cultured in DMEM/F12 medium containing 10% (v/v) DMSO as positive controls. After culturing for 1, 3, and 5 days in a 37°C, 5% CO_2_ incubator, add 10 μL of CCK‐8 reagent to each well and measure the absorbance (*A*) value at 450 nm using a microplate reader.

#### 2.3.2 Scanning electron microscope of scaffold‐cell and live‐dead cell staining

After sterilization, the P3 generation MFCs were dispersed to prepare a single‐cell suspension with a cell concentration of 1 × 10^6^ cells/mL. Squaring scaffold samples with dimensions of 8 mm × 8 mm × 2 mm were prepared, and 100 μL of cell suspension was added dropwise for in vitro culture. 1. Scanning electron microscopy observation: On day 1, the scaffold material loaded with cells was fixed with 2.5% glutaraldehyde, dehydrated sequentially with different concentrations of ethanol, and then freeze‐dried. After sputtering gold on the sample, SEM was used to observe the adhesion and proliferation of cells on the scaffold material. 2. Dead/live cell staining: On day 7, cells grown on the scaffold material were fluorescently stained, rinsed in PBS buffer, and then treated with a dead/live staining reagent (containing 2 μM calcein AM and 4 μM EthD‐1). After rinsing three times with PBS, the samples were observed using a laser confocal microscope to evaluate the ability of the PCL/COL I scaffold to promote cell proliferation. Inoculate 2 × 10^5^ MFCs onto the scaffold samples (8 mm × 8 mm × 2 mm), and collect samples after 1, 4, 7, and 14 days of in vitro culture. Extract DNA from the scaffold‐cell complexes using the TIANamp Genomic DNA Kit, and then precisely measure the DNA using the PicoGreen fluorescence method.

#### 2.3.3 Determination of DNA, collagen, and glycosaminoglycan (GAG) content in scaffold‐cell

Inoculate 2 × 10^5^ MFCs onto the scaffold samples (8 mm × 8 mm × 2 mm), and collect samples after 1, 4, 7, and 14 days of in vitro culture. Extract DNA from the scaffold‐cell complexes using the TIANamp Genomic DNA Kit, and then precisely measure the DNA using the PicoGreen fluorescence method. Measure the collagen content and GAG content in the scaffold before and after cell seeding using the hydroxyproline method and dimethylmethylene blue (DMMB) colorimetric assay. Replace the culture medium of the scaffold‐cell complex every 2 days, collect the culture medium, and store it in a centrifuge tube at −80°C. Since cells secrete collagen and GAG into the culture medium, quantitative determination of collagen and glycosaminoglycans in the culture medium is performed.

### 2.4 Animal experiments

The animal study was approved by the Animal Care Committee of Chinese PLA General Hospital and adhered to the ARRIVE guidelines.

#### 2.4.1 Establishment of animal model and grouping

**FIGURE 2:**
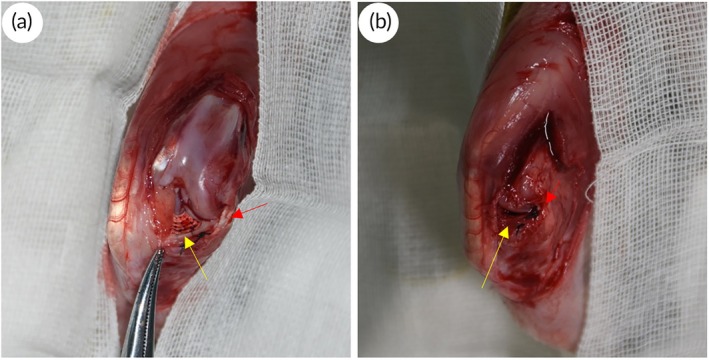
Intraoperative picture of meniscus scaffold implantation in the knee joint of animals (yellow arrow: Meniscus scaffold, red arrow: Medial collateral ligament).

Select 40 healthy New Zealand male white rabbits aged 4–6 months with a body weight of approximately 3 kg and mature skeletons, and randomly divide them into 4 groups (Sham‐operated, PCL/COL I scaffold group, PCL scaffold group, blank defect group). Each rabbit was anesthetized by intramuscular injection of 160 mg ketamine and 12 mg xylazine. The knee joint was flexed, and a medial patellar approach was taken to open the joint capsule and turn the patella outward. Then, the soft tissues surrounding the medial meniscus were bluntly separated, while the medial collateral ligament was completely preserved to fully expose the medial meniscus. The medial 5/6 of the meniscus was removed, leaving only the outer edge and anterior and posterior corners, to construct a model of medial meniscus defect in rabbits.^24^ The surgical approach adopted in this experiment can completely preserve the medial collateral ligament and the cruciate ligament, which is crucial for maintaining the stability of the knee joint (Figure 2).

Sham‐operated: no treatment was performed after opening the joint capsule; PCL/COL I scaffold group: PCL/COL I meniscus scaffold was implanted; PCL scaffold group: 100% PCL meniscus scaffold was implanted; blank defect group: no further treatment was performed after successful modeling. After surgery, intramuscular injection of penicillin was administered for 3 days to prevent infection. The rabbit's knee joint was left in a freely movable state postoperatively. At two time points, namely three and 6 months postoperatively, the animals were euthanized for sampling to observe and evaluate the regeneration and repair of the meniscus as well as the damage to the knee joint cartilage.

#### 2.4.2 Gross morphology and histological evaluation of meniscus

After separating the femur, tibial plateau, and newly formed meniscus tissues, photographs were taken. Gross observations and assessments were conducted on the morphology, smoothness, and size of the newly formed meniscus tissues, as well as the smoothness and damage of the cartilage on the tibial plateau and femoral condyle.

After dehydration, paraffin embedding, and sectioning of the meniscus tissue, hematoxylin eosin (HE) staining, saffron O/ fast green staining (SO), toluidine blue staining (TB), and picrosirius red (PR) staining were performed. Histological evaluation of the regenerated meniscus was conducted, and Ishida scoring was used for semi‐quantitative histological evaluation of the regenerated meniscus.^31^ Additionally, immunohistochemistry staining was performed to label type I and type II collagen. After decalcifying the tibial plateau and femoral condyle tissues with 10% EDTA decalcifying solution for 7 weeks, dehydration, paraffin embedding, and sectioning were performed. Subsequently, HE, SO, and TB staining were performed, and light microscopy was used to observe and evaluate the cartilage damage of the tibial plateau and femoral condyle.

#### 2.4.3 Imaging assessment and biomechanical testing of meniscus

Using a small animal x‐ray imaging system, we obtained the anterior–posterior and lateral views of the rabbit knee joint. By comparing indicators such as joint cavity space and osteophyte formation in each group, we assessed the severity of arthritis. Additionally, we evaluated the degenerative changes in the rabbit knee joint using the Kellgren–Lawrence (K–L) grading scale.^31^

Cut the meniscus tissues of each group of newborns into tensile mechanical test samples of uniform specifications. During tensile testing, a 5% pre‐stretch is performed at a rate of 5 mm/min. After 12 cycles of stretching, the sample is stretched to fracture at a rate of 5 mm/min, obtaining a stress–strain curve. The tensile elastic modulus is then calculated.

### 2.5 4Molecular biology evaluation

*Quantitative detection of GAG*: Isolate the newborn meniscus tissue, rinse it three times with PBS buffer, and freeze‐dry it for 24 h. Then, accurately weigh 20 mg of the newborn meniscus tissue and detect the GAG content using the dimethylmethylene blue (DMMB) colorimetric assay.

*Quantitative detection of collagen*: According to the above method, accurately weigh 50 mg of newborn meniscus tissue and use a hydroxyproline quantitative assay kit to determine the collagen content of the newly formed meniscus tissue.

### 2.6 Statistical methods

All statistical analyses were performed using SPSS v. 25.0 (IBM Corporation; Armonk, New York, USA). For normally distributed data, quantitative data were subjected to one‐way analysis of variance or Student's *t*‐test, whereas nonparametric Kruskal–Wallis tests were used for skewed data. Statistical significance was set at a two‐sided *p*‐value of <0.05.

## 3 RESULTS

### 3.1 Observation of scaffold morphology and microstructure

**FIGURE 3:**
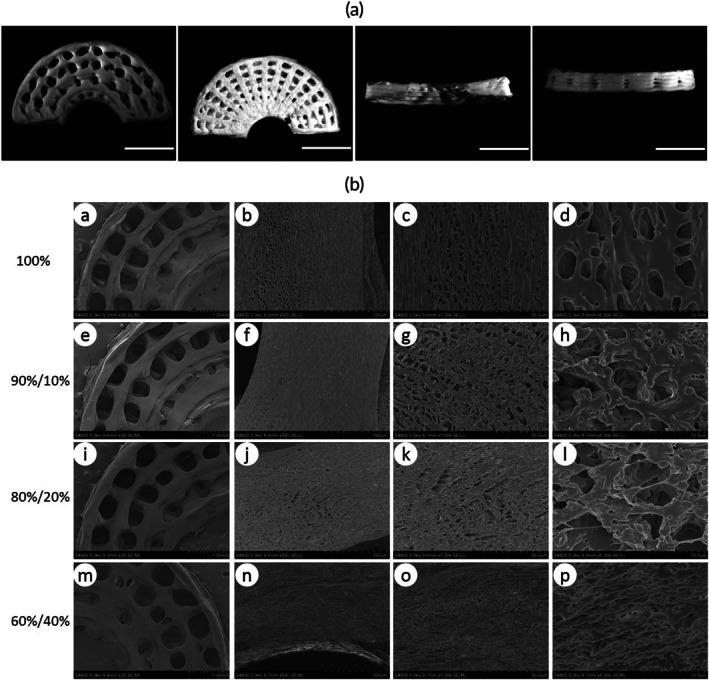
General view of PCL/COL I integrated meniscus scaffold (a). PCL /COL I integrated meniscus scaffold scanning electron microscopy observation from left to right were enlarged by 30, 500, 1000, and 5000 times, respectively (b). PCL meniscus scaffold (a′)–(d′). 90%PCL/10%COL I meniscus scaffold (e′)–(h′). 80%PCL/20%COL I meniscus scaffold (i′)–(l′). 60%PCL/40%COL I meniscus scaffold (m′)–(p′).

The morphology of the PCL/COL I meniscus scaffold presents a circular radial wedge shape (Figure 3a). Under low‐magnification scanning electron microscopy, the scaffold appears as a multilayered network structure. Under high magnification, dense irregular pores can be observed on the scaffold structure, and as the COL I component in the meniscus scaffold increases, the surface roughness of the scaffold also increases (Figure 3b).

### 3.2 Scaffold composition analysis

**FIGURE 4:**
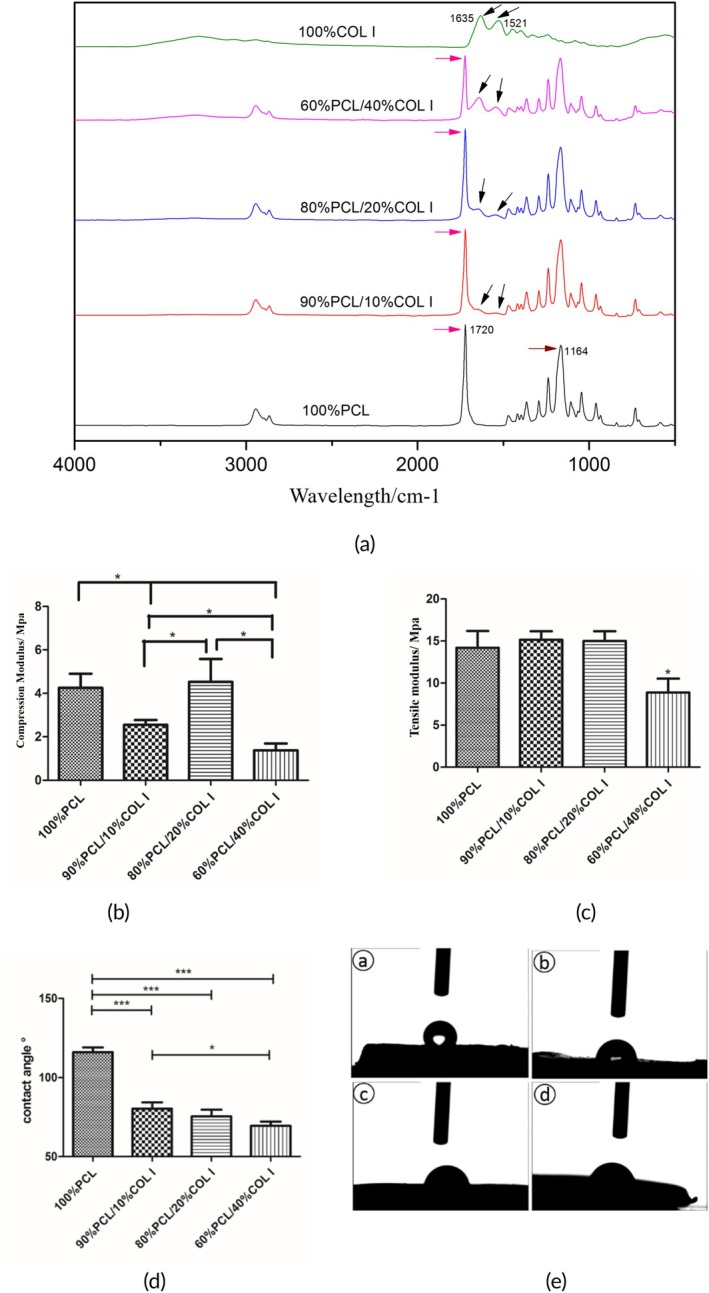
Infrared spectra of PCL/COL I scaffold materials with different mass ratios (a, *n* = 2). Detection results of the biomechanical properties of PCL/COL I scaffolds with different mass ratios. Meniscal scaffolds compression test (b, *n* = 3). Meniscal scaffolds tensile test (c). Surface contact Angle detection results of each group of scaffold materials (d, *n* = 3). The falling image of deionized water (e). 100%PCL (a′), 90%PCL/10%COL I (b′), 80%PCL/20%COL I (c′), 60%PCL/40%COL I (d′). (*represent *p* < 0.05, ** represent *p* < 0.01, *** represent *p* < 0.001).

The results of FTIR analysis indicate that PCL and COL I exhibit distinct characteristic peaks (Figure 4a). The PCL meniscus scaffold exhibits a carbonyl characteristic peak (pink arrow) at approximately 1720 cm^−1^, as well as a C−O characteristic peak (brown arrow) at approximately 1164 cm^−1^. The COL I meniscus scaffold exhibits characteristic peaks at 1635 and 1521 cm^−1^ (indicated by black arrows), which may correspond to the characteristic peaks of C–N bonds and N–H bonds. The PCL/COL I meniscus scaffold possesses characteristic peaks of both PCL and COL I. As the content of COL I in the scaffold increases, the intensity of the characteristic peak of COL I increases correspondingly, while the intensity of the characteristic peak of PCL decreases relatively.

### 3.3 Mechanical property testing results of the scaffold

In the compression test, the compressive modulus of both the 90% PCL/10% COL I group and the 60% PCL/40% COL I group was significantly lower than that of the 100% PCL group (4.258 ± 0.645 Mpa) (the difference was statistically significant, *p* < 0.05). Interestingly, there was no statistically significant difference in the compressive modulus (4.529 ± 1.051 Mpa) between the 80% PCL/20% COL I group and the 100% PCL group. This may be caused by the combination of chemical bonds between PCL and COL I when they are mixed in a mass ratio of 80:20, which alters the crystallinity, grain size, and crystal structure (Figure 4b).

In the tensile test, the tensile modulus of the 60% PCL/40% COL I group (8.875 ± 1.653 Mpa) was significantly lower than that of the remaining three groups, with the difference being statistically significant (*p* < 0.05). There was no significant difference in tensile modulus among the other three groups (*p* > 0.05). The decrease in tensile modulus of the 60% PCL/40% COL I scaffold may be related to the significant reduction in PCL content per unit volume (Figure 4c).

### 3.4 Hydrophilicity testing of scaffolds

It is generally believed that the surface of hydrophilic materials is more conducive to cell adhesion and growth.^32^ The surface contact angle of the 100% PCL scaffold is 116.08 ± 2.99°. As the COL I content increases, the surface contact angle of the scaffold tends to decrease. The surface contact angle of the 60% PCL/40% COL I scaffold is the smallest, at 69.51 ± 2.71°. However, when compared to the 80% PCL/20% COL I scaffold (75.49 ± 4.20°), the difference is not statistically significant (*p* > 0.05) (Figure 4d, e). During the experiment, water droplets will be absorbed by the PCL/COL I scaffold material, but not by the PCL scaffold material. The test indicated that COL I can enhance the hydrophilicity of PCL/COL I scaffolds, and the hydrophilicity of 60% PCL/40% COL I and 80% PCL/20% COL I scaffolds is relatively good.

### 3.5 Cytotoxicity assessment

**FIGURE 5:**
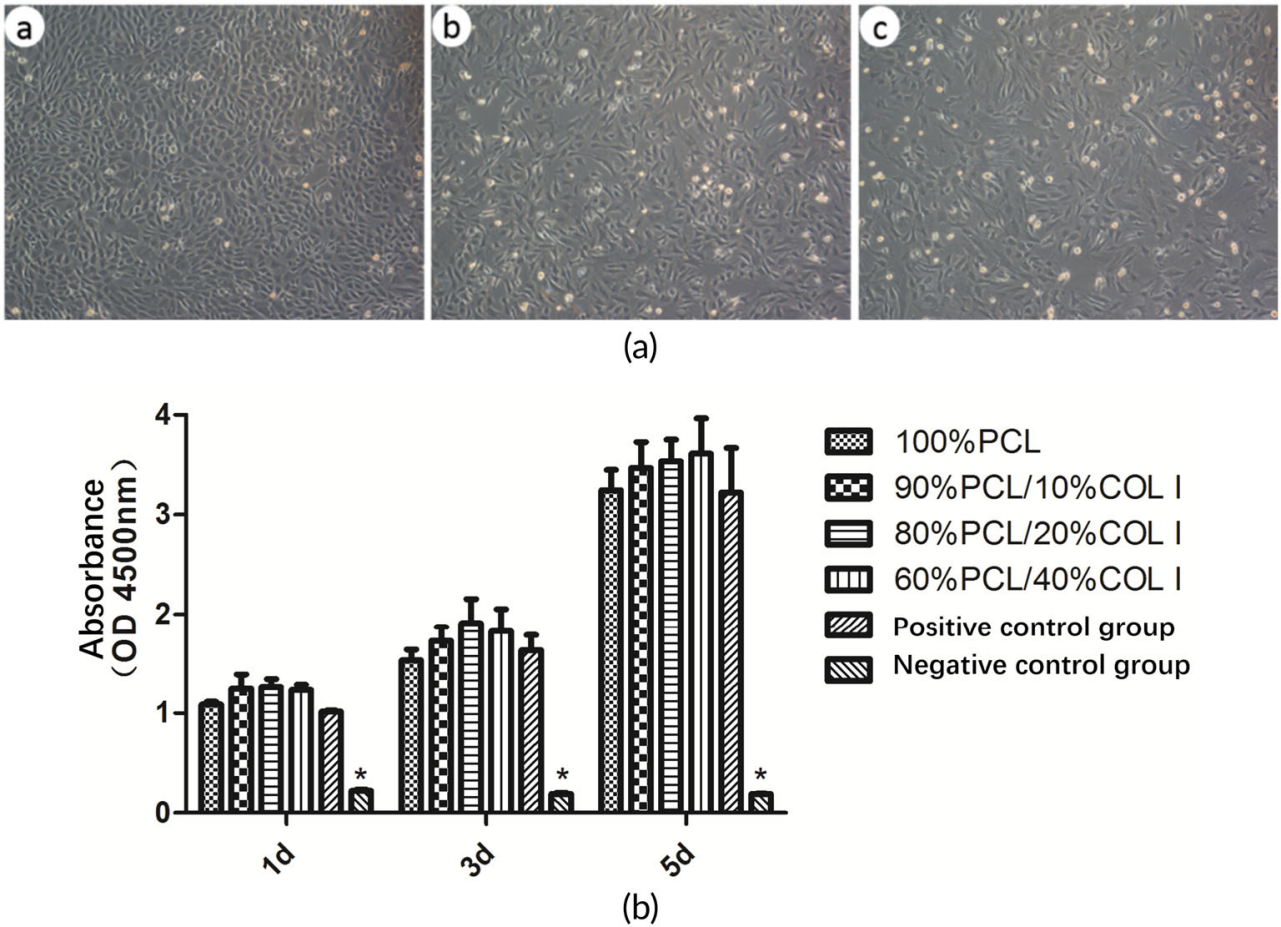
In vitro cell culture results (a). P0 generation (a′), P1 generation (b′) and P3 generation (c′). Cytotoxicity of PCL/COL I scaffolds with different mass ratios (b, *n* = 5).

The MFCs cultured in vitro are shown in Figure 5a. The MFCs in the P0 generation are round or nearly round, while with the increase in passaging times, the morphology of MFCs becomes spindle‐shaped or polygonal, indicating that MFCs gradually lose their cell phenotype during in vitro planar culture. Over time, the number of cells cultured in the leaching solution of PCL/COL I scaffolds showed an increasing trend. There was no statistically significant difference in OD450 values between each time point and the normal group (negative control group) (*p* > 0.05), indicating that the extracting solution of the scaffolds has no cytotoxicity and exhibits good cell compatibility (Figure 5b).

### 3.6 Scanning electron microscopy and live‐dead Cell staining of scaffold‐cell complexes

**FIGURE 6:**
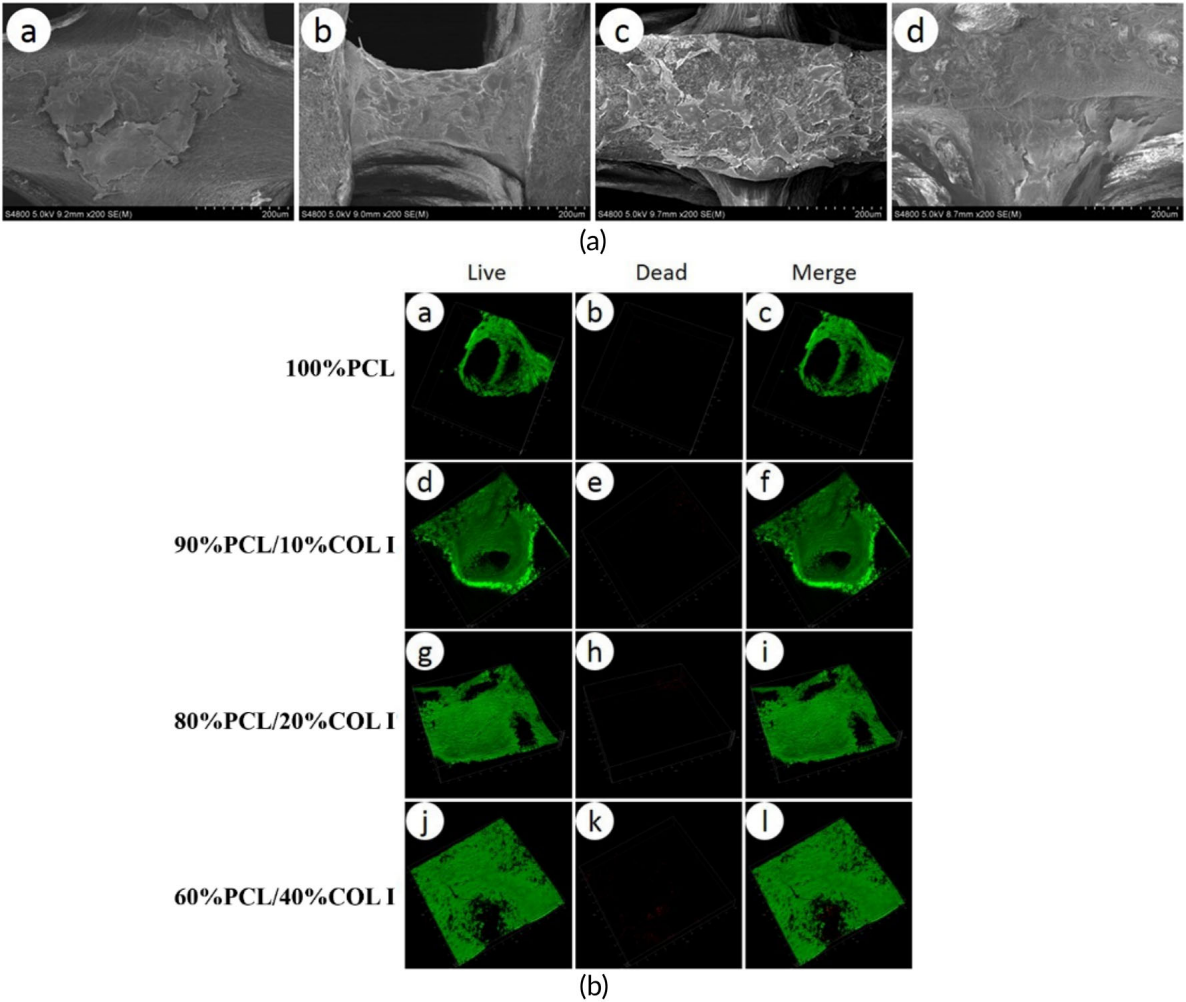
SEM results of scaffold‐cell complex (a) 100%PCL scaffold (a′), 90%PCL/10%COL I scaffold (b′), 80%PCL/20%COL I scaffold (c′), D 60%PCL/40%COL I scaffold (d′). Live‐dead staining results of four PCL/COL I scaffold cell complexes with different mass ratios (b, *n* = 3).

Scanning electron microscopy observations revealed that MFCs could adhere and proliferate on the surfaces of four types of scaffolds (Figure 6a). The surfaces of the 60% PCL/40% COL I scaffold and the 80% PCL/20% COL I scaffold were more prone to cell adhesion, while the 100% PCL scaffold had fewer adhering cells.

As shown in Figure 6b, a large number of viable MFCs (green fluorescence) were visible on all four types of scaffolds, while only a very small number of dead cells (red fluorescence) were observed. Among them, a large number of proliferating MFCs were visible on the 80% PCL/20% COL I and 60% PCL/40% COL I scaffolds, maintaining high cell activity.

### 3.7 Molecular biological detection of scaffold‐cell complexes

**FIGURE 7:**
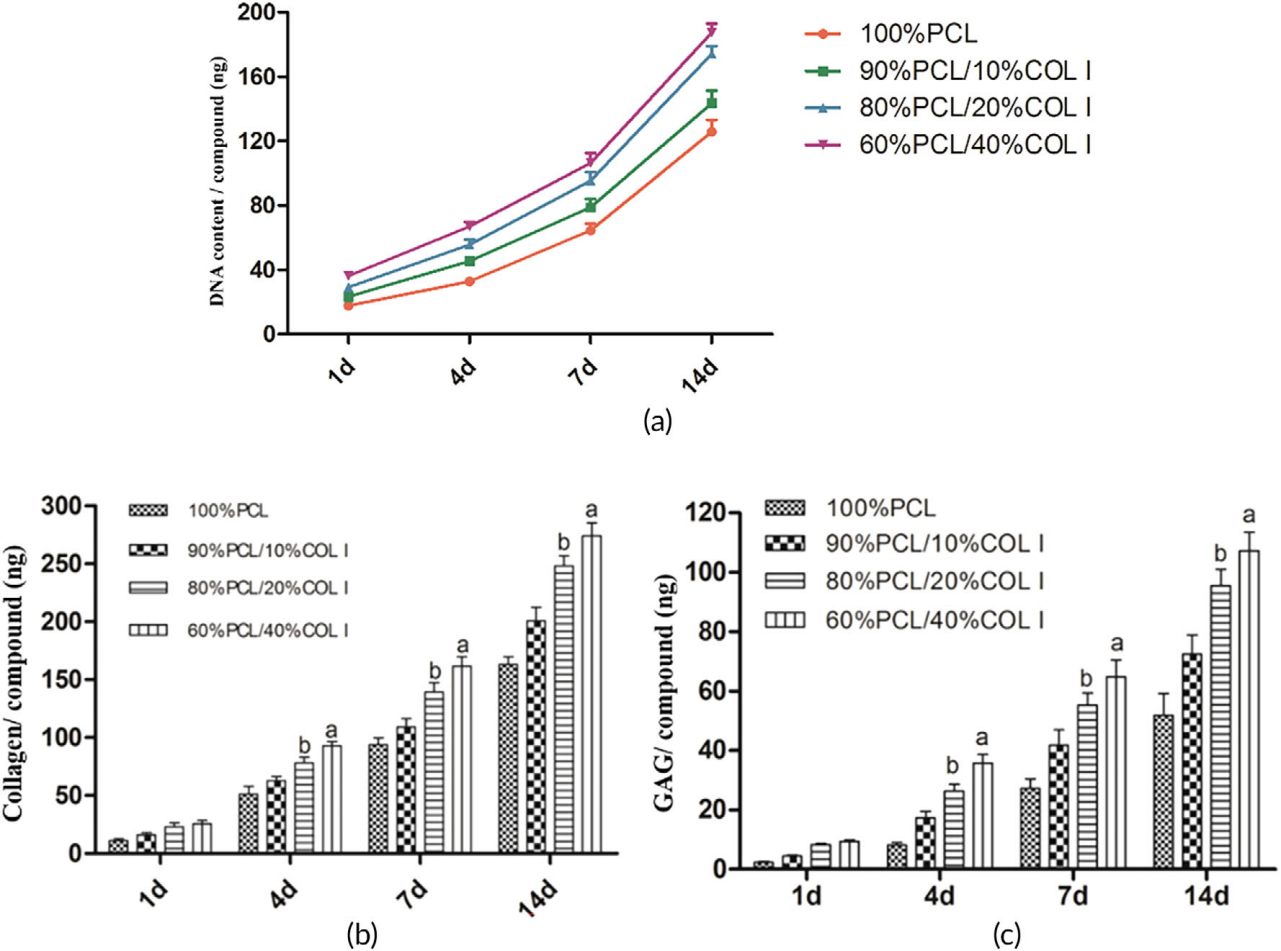
Results of quantitative experiment of scaffold cell complex DNA (a, *n* = 5). Quantitative detection results of collagen and glycosaminoglycan (b and c, *n* = 5).

As shown in Figure 7, with the passage of culture time, DNA content, collagen content, and GAG content all exhibited an increasing trend, indicating that MFCs can proliferate on all four types of scaffolds and can promote the secretion of collagen and GAG by MFCs. The DNA, collagen, and GAG content secreted by the 60% PCL/40% COL I and 80% PCL/20% COL I groups were higher than those of other groups, with statistically significant differences (*p* < 0.05). However, there was no significant difference between the two groups (*p* > 0.05).

### 3.8 Animal experiments

Based on the experimental results mentioned above, the 80% PCL/20% COL I meniscus scaffold, which possesses both excellent biomechanical properties and good cellular compatibility, has been selected for further animal experiments.

#### 3.8.1 Gross evaluation of knee joint tissue

**FIGURE 8:**
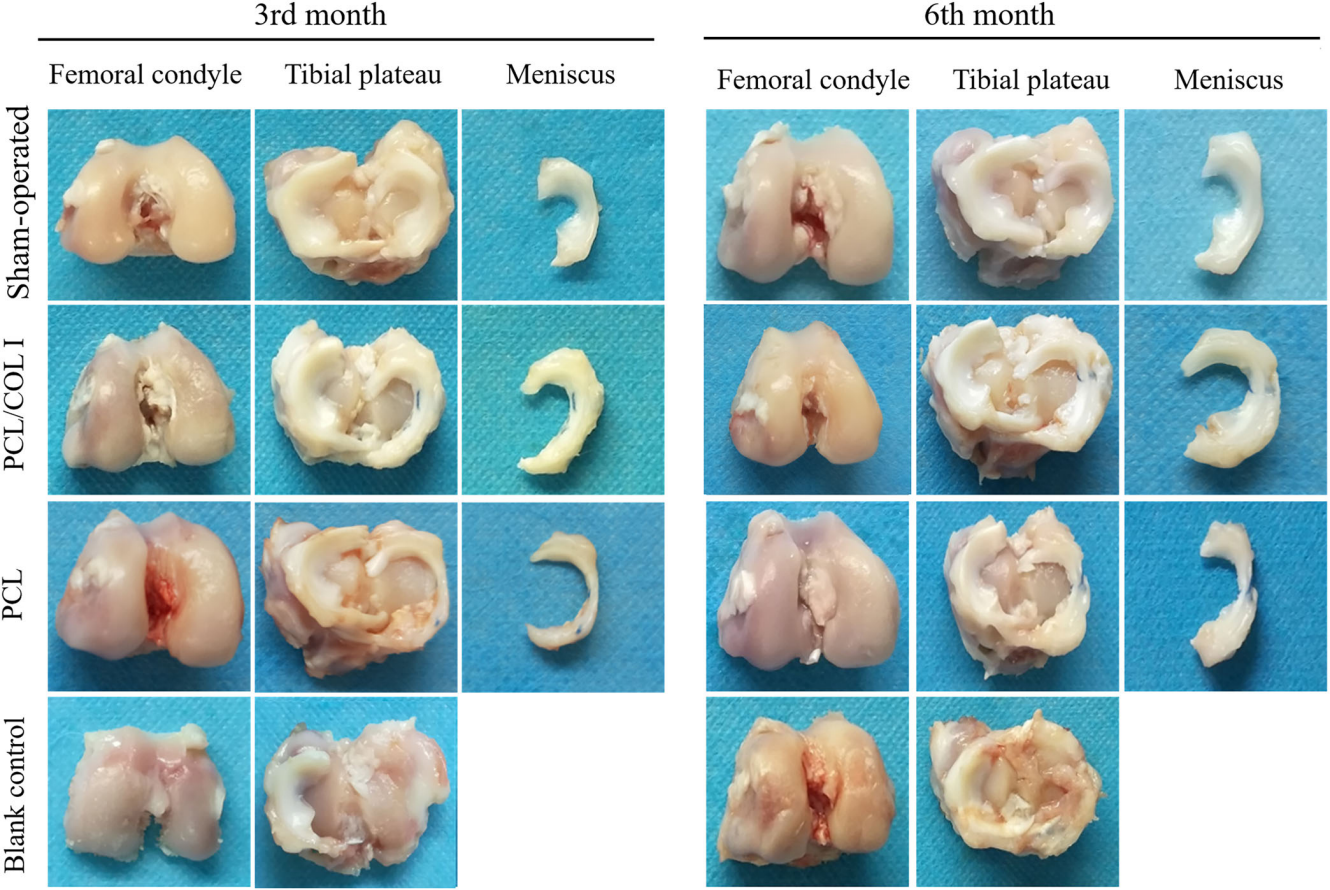
General view of regeneration and repair of meniscus tissue and articular cartilage (*n* = 5 for each time point).

Separate the meniscus tissue, femoral condyle, and tibial plateau from the rabbit's knee joint, and observe their gross structural morphology (Figure 8). Three months post‐surgery, crescent‐shaped meniscus‐like tissue formation was observed in the PCL/COL I group, with smooth cartilage surfaces on the femoral condyle and tibial plateau, and no significant wear was noted. In the PCL scaffold group, crescent‐shaped meniscus‐like tissue formation was observed, but compared to the PCL/COL I group, the volume of the newly formed meniscus‐like tissue was significantly reduced. In the blank control group, no meniscus‐like tissue formation was observed, with only a small amount of synovial tissue ingrowth. Additionally, there was moderate wear on the cartilage surface of the femoral condyle and tibial plateau, and osteophytosis formation due to bone hyperplasia.

Six months post‐surgery, the newly formed meniscus tissue in the PCL/COL I group was larger than that at 3 months, and its morphology resembled that of a natural meniscus. There was slight wear on the cartilage of the femoral condyle and tibial plateau. The PCL group is similar to the PCL/COL I group, but the meniscus formed in the PCL group is smaller than that in the PCL/COL I group. The wear and tear of the femoral condylar cartilage and tibial plateau cartilage in the blank control group further aggravated, with an increase in osteophytes, exhibiting manifestations of osteoarthritis.

#### 3.8.2 Histological evaluation of meniscus

**FIGURE 9:**
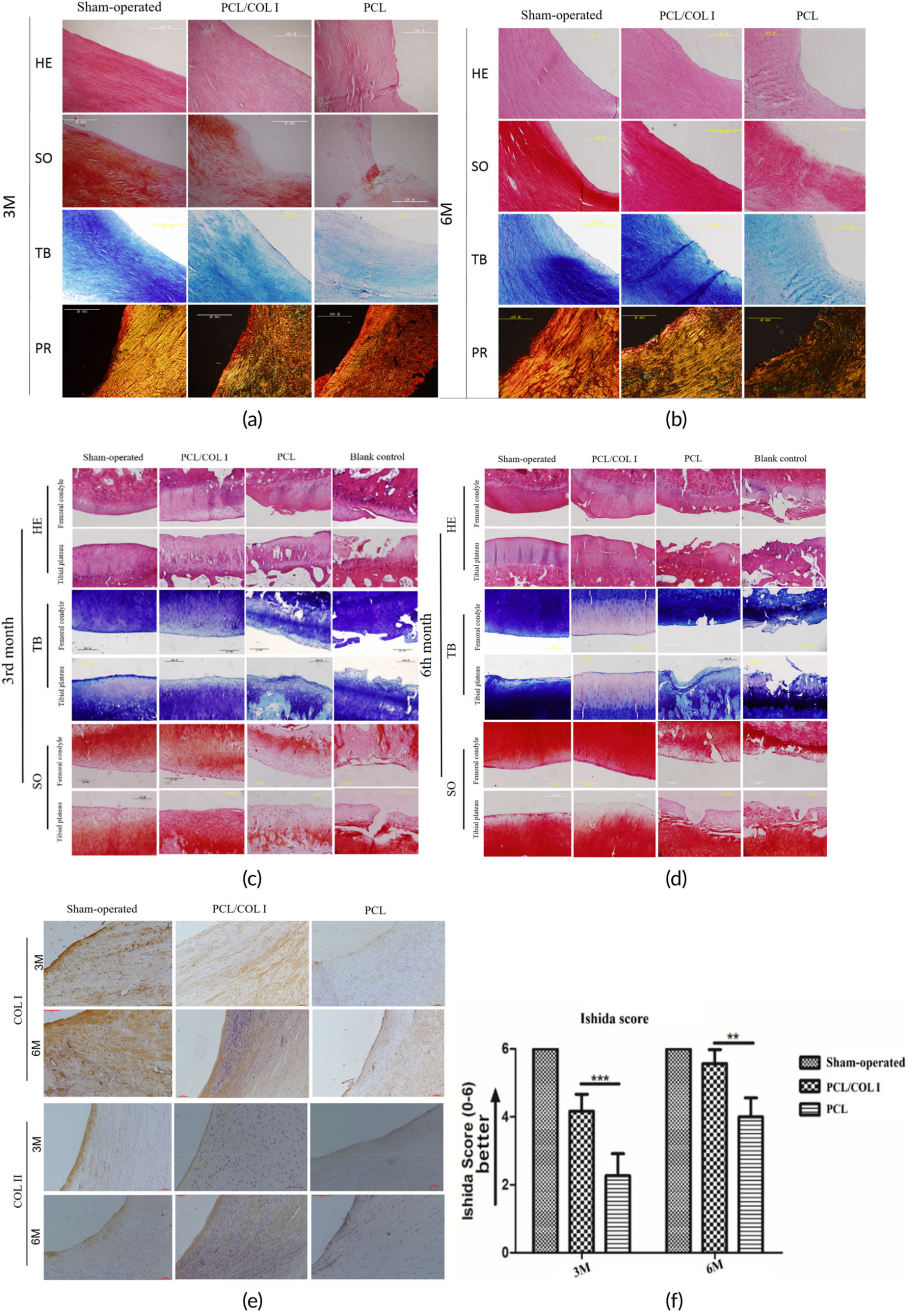
Histological staining of regenerated meniscus at 3 and 6 months after surgery (a), (b). HE staining of femoral condyle and tibial plateau cartilage (c), (d). Immunohistochemical results of COL1 and COL II (e). Histological score of regenerated meniscus tissue (f, *n* = 3 for each time point) (** represent *p* < 0.01, *** represent *p* < 0.001).

The staining results of meniscus tissue are shown in Figure 9 a, b. The HE staining results revealed that in the PCL/COL I group, the fibrocartilage structure of the meniscus was observable, and uniformly arranged chondroid cells were visible within the newly formed meniscus tissue. At 6 months, the newly formed meniscus tissue exhibited more cartilage lacunae structures, and the fibrous arrangement was more regular compared to that at 3 months. The newly formed meniscus tissue in the PCL group contained a small number of scattered chondrocyte‐like cells, with fewer cartilage lacunae structures, and the fiber arrangement was not as neat as that in the PCL/COL I group.

At 3 months, the newly formed meniscus tissue in the PCL/COL I group showed positive staining with Saffron O/fast green staining (SO). At 6 months, the newly formed meniscus tissue exhibited strong positive staining. Furthermore, there was no significant difference in the staining intensity of the newly formed meniscus between these two time points and the sham surgery group. The staining intensity of SO in the PCL group is lighter than that in the PCL/COL I group. At 3 months, the newborn meniscus tissue in the PCL/COL I group exhibited positive TB staining. At 6 months, the staining became strongly positive. The staining intensity of the newborn meniscus at both time points was comparable to that of the sham surgery group, and lacunae structures were visible in both cases. The PR of the fresh meniscus tissue in the PCL/COL I group revealed neatly arranged and regularly shaped collagen fibers. At 6 months, the collagen fibers were more neatly and regularly arranged than at 3 months, showing no significant difference from the sham surgery group and significantly outperforming the PCL group.

Using the Ishida scoring system to conduct a histological semi‐quantitative evaluation of the new meniscus in each group, the results showed that the PCL/COL I group had significantly better scores than the PCL group (*p* < 0.05, statistically significant) (Figure 9f). In the immunohistochemical results of COL I and COL II, it was observed that the staining intensity of both PCL/COL I and PCL groups increased over time. Furthermore, the staining intensity of both types of collagen in the PCL/COL I group was higher than that in the PCL group. Additionally, the staining intensity of COL II was higher than that in the sham surgery group, while the staining intensity of COL I was close to that in the sham surgery group (Figure 9e).

#### 3.8.3 Histological evaluation of femoral condyle and tibial plateau

Evaluate the wear condition of articular cartilage by conducting HE, SO, and TB staining on the femoral condyle and tibial plateau (Figure 9c, d). In the PCL/COL I group, the femoral condylar cartilage surface was smooth at 3 months and remained mostly smooth at 6 months while the tibial plateau cartilage surface exhibited mild wear. At 3 months, the femoral condylar cartilage surface in the PCL group exhibited mild wear. At 6 months, the femoral condylar cartilage surface showed aggravated damage, presenting moderate wear. Additionally, the tibial plateau cartilage surface exhibited cartilage cracks, unevenness, and thinner cartilage thickness, presenting moderate wear. The femoral condyle and tibial plateau cartilage in the blank defect group exhibited severe damage, with a rough surface, very thin cartilage thickness, and defects. In some areas, subchondral bone exposure was visible. Under TB and SO staining, the staining intensity of each group increased with time. However, in the PCL/COL I group, there was a distinct light blue cartilage formation area in the TB staining at 6 months compared to other groups. In the SO staining, the staining intensity of the PCL/COL I group was also significantly higher than that of the PCL group and the blank defect group and was similar to that of the sham surgery group.

#### 3.8.4 Imaging evaluation

**FIGURE 10:**
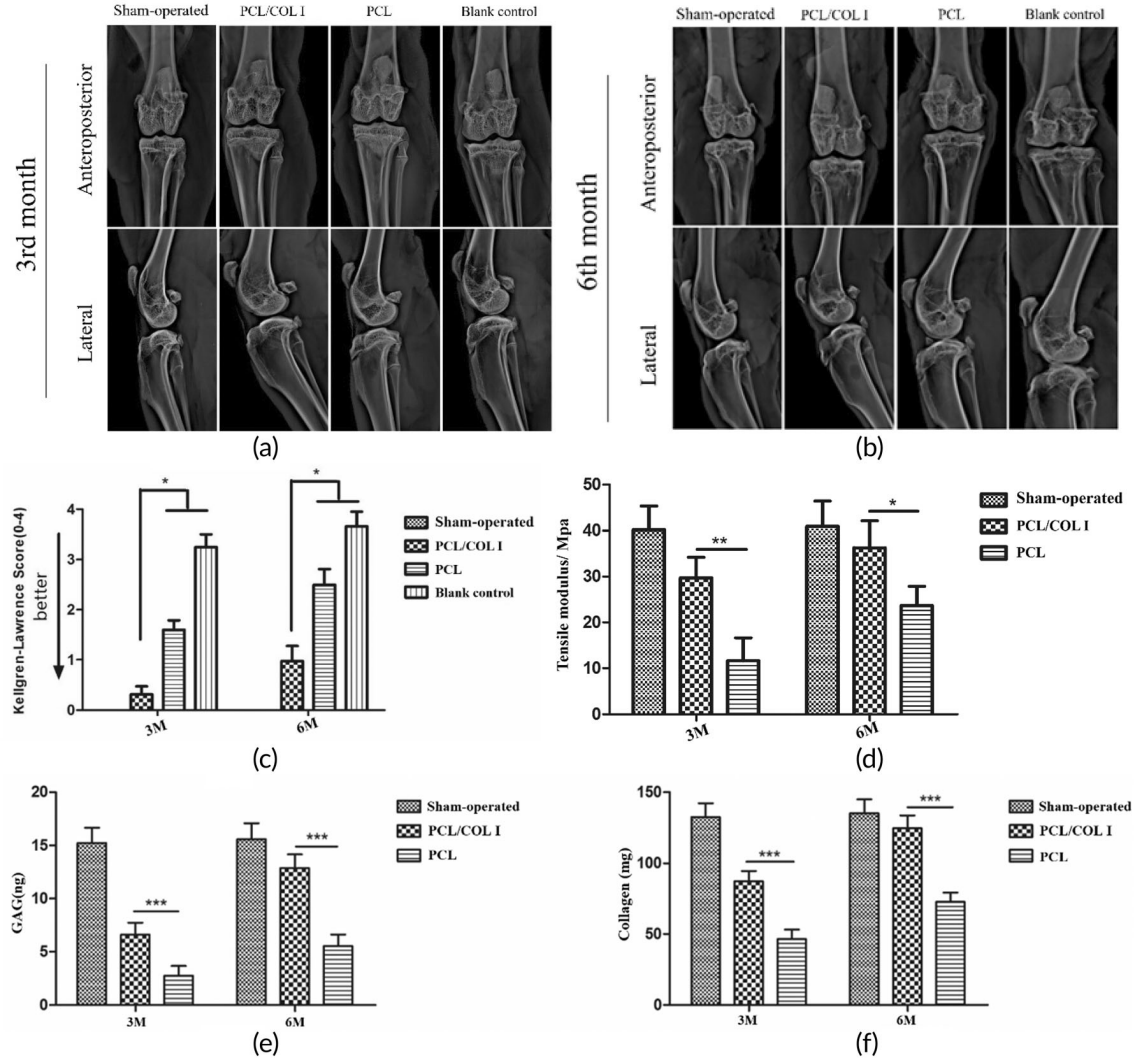
Results of x‐ray examination of the knee joint (a), (b). Knee x‐ray K–L rating (c, *n* = 5 for each time point). Results of biomechanical properties of newborn meniscus tissue (d, *n* = 3). Results of quantitative analysis of glycosaminoglycan and collagen in neo‐meniscus tissue (e, f, *n* = 3). (*represent *p* < 0.05, ** represent *p* < 0.01, *** represent *p* < 0.001).

In the PCL/COL I group, the knee joint x‐ray appearance was normal, with no narrowing of the joint cavity, no subchondral bone cysts or sclerosis, and no osteophyte formation. The joint cavity space in the PCL group showed mild narrowing, with a rough and uneven joint surface, and slight osteophytosis visible around the medial tibial plateau. In the later stage, the joint cavity space narrowed significantly; the roughness of the joint surface and osteophytosis of the medial tibial plateau further aggravated. The joint cavity of the blank control group exhibited significant narrowing, with rough and uneven articular surfaces (Figure 10a–c). Additionally, subchondral sclerosis of the medial tibial plateau and peripheral osteophyte formation were visible. The knee joint was evaluated using the K–L grading scale, and the results indicated that the PCL/COL I scaffold group scored significantly better than the other groups(*p* < 0.05, statistically significant).

#### 3.8.5 Molecular biological evaluation and biomechanical evaluation

Over time, the GAG and collagen content in each group gradually increased (Figure 10e, f). The GAG and collagen contents in Group PCL/COL I were significantly higher than those in Group PCL, with a statistically significant difference (*p* < 0.05), and were close to those in the sham surgery group. As shown in Figure 10d, the tensile modulus of each group in June was higher than that in March. The tensile modulus of the PCL/COL I group was higher than that of the PCL group, with no significant difference compared to the sham surgery group.

## 4 DISCUSSION

Due to the special anatomical structure of the meniscus, only 10%–25% of the area around it has blood supply. Therefore, once the meniscus is damaged, its self‐repair ability is extremely limited. For meniscus injury, clinical treatment methods (such as symptomatic pain relief, suture, resection, allogeneic meniscus replacement, etc.) have certain limitations. Therefore, regenerative repair of meniscus injury remains a challenging scientific problem.^33^, ^34^, ^35^ In recent years, tissue engineering technology has developed rapidly, and scholars at home and abroad believe that it is expected to solve the challenge of regeneration and repair after meniscus injury. The key elements of tissue engineering include scaffolds, seed cells, and growth factors, and the selection of appropriate materials is essential for the performance of scaffolds. At present, the scaffold materials of tissue‐engineered meniscus are mainly divided into: 1. Synthetic polymer materials; 2. Tissue‐derived scaffold materials; 3. Biological materials. Synthetic polymer materials mainly include: polylactic acid (PLA), polyglycolic acid, polylactic acid‐glycolic acid copolymer (PLGA), polyurethane (PU), PCL, polyvinyl alcohol, poly L‐lactic acid (PLLA).^36^, ^37^, ^38^, ^39^ Biological materials mainly include: fibrin, silk fibroin, collagen, agarose, chitosan (CS), alginate, gelatin, methyl methacrylate gelatin (Gel‐MA).

However, a large number of studies have reported that it is difficult for a single material to meet the requirements of meniscus tissue engineering scaffolds at the same time: good biocompatibility and biomechanical properties, controlled degradation, excellent plasticity, and unlimited sources.^40^, ^41^, ^42^, ^43^ PCL has good mechanical properties and degradability, but its limitations include low cellular affinity and relatively poor biocompatibility. Nevertheless, extensive research has been conducted to overcome the limited cytocompatibility of PCL through its combination with other highly biocompatible materials, demonstrating significant improvements in cellular interactions. Shuang Gao et al. combined the decellularized meniscus extracellular matrix (DMECM) with PCL via electrospinning to fabricate scaffolds that matched the mechanical properties of the natural meniscus, while the incorporation of DMECM effectively promoted the proliferation of meniscus cells.^44^ Hao Li et al. presented a composite scaffold by 3DP a PCL scaffold as a backbone, followed by injection with the meniscus extracellular matrix (MECM), and modification with kartogenin (KGN)‐loaded poly(lactic‐co‐glycolic) acid (PLGA) microsphere (mS), which serves as a drug delivery system.^45^ The PCL/MECM‐KGN μS scaffold not only inherits the excellent physical properties of PCL materials, but also exhibits superior cell affinity and cell vitality preservation, as well as chondrogenic activity.

The ECM of the meniscus is mainly composed of proteoglycans and collagen fibers. Type I collagen (COL I) itself is the main component of the ECM of fibrocartilage, promoting the adhesion, proliferation, and differentiation of chondrocytes, and assisting chondrocytes to eventually form fibrocartilage.^46^, ^47^, ^48^ Gaëlle Savin et al. fabricated a COL I/poly(ester‐urethane) (PEU) meniscus scaffold by integrating COL I with PEU. In comparison to a standalone PEU meniscus scaffold, COL I not only offers superior cell affinity but also enhances cell proliferation and sustains the cellular microenvironment.^49^ Therefore, focusing on the in situ regeneration of damaged knee meniscus, we propose a hybrid meniscus scaffold composed of PCL and COL I, which combines suitable biomechanical properties with biocompatibility.

Most of the collagen fibers in the meniscus are arranged in a circular manner, and a small part of them is arranged radially.^50^ Therefore, the meniscus is a highly anisotropic tissue, which has led to the widespread use of 3DP technology in the preparation of tissue‐engineered meniscus scaffolds. Compared with traditional scaffold manufacturing technology, 3DP technology has the advantages of good repeatability, controllable microstructure, and shape of scaffolds. Bas van Bochove^51^ used polytrimethylene carbonate as raw material to make goat meniscus implants by stereolithography (SLA). By adjusting the pore size and porosity, he was able to make the mechanical properties of the scaffold close to those of the natural meniscus. Zhang et al.^52^ used fused deposition manufacturing (FDM) technology to fabricate meniscus scaffolds with different pore sizes. In vitro and in vivo experiments showed that the average pore size of 3d printed PCL scaffolds could significantly affect cell behavior and meniscus repair outcomes. Yang et al.^53^ made an artificial meniscus with a high degree of anisotropy using electronically assisted 3D printing. These studies show that 3DP can precisely control the microstructure inside a structure and even simulate some anisotropic structures using improved 3DP techniques.

Low‐temperature deposition manufacture‐3DP technology can achieve high‐precision printing of PCL and collagen type I meniscus scaffolds without damaging collagen type I and maximize the biological activity of the materials. In this study, the PCL/COL I bionic meniscus scaffold was constructed by low‐temperature deposition 3DP technology, and the composition and microstructure were double bionic to simulate the natural meniscus.

The PCL/COL I scaffolds with different mass ratios prepared in this experiment have a porous structure, which is conducive to the delivery of nutrients and promotes cell adhesion and proliferation, providing a network scaffold for tissue generation.^54^, ^55^ According to literature reports,^56^, ^57^ the rough surface of materials can increase the contact area between cells and materials, thereby promoting cell adhesion and proliferation. In this experiment, with the increase of COL I content, the surface roughness of the scaffold increased and the contact Angle of the material decreased continuously, indicating that COL I can significantly enhance the cytocompatibility and hydrophilicity of the PCL/COL I meniscus scaffold. Ebrahimi Z et al.^58^ also found that the contact Angle decreased after PCL was combined with collagen. Arango‐Santander et al.^59^ found that under normal conditions, the surface of hydrophilic materials is more conducive to cell adhesion and growth. Cytotoxicity experiments showed that PCL/COL I scaffolds had good biocompatibility and could promote the proliferation of MFCs, increase the secretion of ECM (glycosaminoglycans and collagen), and simulate the microenvironment of MFCs growth, thereby maintaining the phenotype of MFCs. Moreover, the higher the content of COL I in the scaffolds, the stronger the proliferation and differentiation of MFCs. In vitro experiments showed that 80%PCL/20%COL I scaffolds and 60%PCL/40%COLI scaffolds had better biological properties. Combined with the mechanical properties of scaffolds with different mass ratios, the optimal mass ratio of 80%PCL/20%COL I meniscus scaffold was selected to repair meniscus defects in rabbits.

In animal experiments, meniscus regeneration repair in the PCL/COL I group was significantly better than that in the PCL group and blank control group. At 6 months after surgery, the new meniscus was similar to that of the sham group, and the cartilage surface of the femoral condyle and tibial plateau was smooth without obvious wear. There was slight wear on the articular cartilage surface at 6 months, which may be due to the fact that the mechanical properties of the new meniscus have not fully reached the mechanical properties of the natural meniscus during the regeneration of the meniscus tissue and cannot fully exert the function of the meniscus, resulting in slight wear on the articular cartilage surface. In the PCL/COL I group, the new meniscus tissue showed chondroid cells and extracellular matrix. Both SO staining and TB staining were strongly positive, indicating that the nascent meniscus contained large amounts of glycosaminoglycans. PR staining showed that the collagen fibers were arranged neatly and closely. These results confirmed that the newly formed tissue was meniscus tissue. The histological findings of the cartilage of the femoral condyle and tibial plateau were also consistent with the gross observations, and the cartilage surface was smooth. The knee x‐ray films showed that the articular cartilage in the PCL/COL I group was well protected without obvious signs of joint degeneration or osteoarthritis, but the PCL group had mild to moderate articular cartilage damage and osteoarthritis. Glycosaminoglycan and collagen contents in the nascent meniscus were significantly higher in the PCL/COL I group than in the PCL group. In addition, the tensile elastic modulus of the regenerated meniscus in the PCL/COL I scaffold group was also higher than that in the PCL group and close to that in the sham‐operated group. An ideal meniscus tissue engineering scaffold must have excellent biomechanical properties and good biocompatibility.^60^ On the one hand, good mechanical support can offset the stress load from the femur and tibia and maintain the integrity of the scaffold. On the other hand, it can provide ample space for tissue regeneration and cell growth. Good biocompatibility can provide a favorable cellular microenvironment for cell growth and proliferation, promote cell migration, adhesion, proliferation, and differentiation, and thus promote tissue regeneration and maturation. Therefore, in this experiment, it was also verified that the meniscus regeneration repair effect of the PCL scaffold alone group was worse than that of the PCL/COL I scaffold group.

Based on the rabbit meniscus repair model, it can be concluded that the PCL‐COL I scaffold can achieve a certain balance between scaffold degradation and meniscus regeneration. This implies that the degradation rate of the cell‐free PCL‐MECM scaffold may, to some extent, match the in situ regeneration rate of the meniscus in vivo.

Low‐temperature deposition technology (LDM), as an innovative form of 3DP technology, has brought breakthrough progress to the field of regenerative medicine due to its ability to process biological materials under low‐temperature conditions. This printing method avoids the destruction of bioactive molecules (such as growth factors and proteins) caused by high temperatures, preserving their natural structure and function. It significantly enhances the biological activity of the scaffold and demonstrates broad application prospects in the fields of cartilage, meniscus, and nerve regeneration and repair. It can be anticipated that in the future, it will be possible to construct organoids or organ‐on‐a‐chip using a combination of LDM technology and 4D‐printing technology.

However, LDM technology still needs to be optimized in terms of mechanical properties and large‐scale production. Nevertheless, the potential of LDM technology in complex tissue regeneration, personalized treatment, and in situ repair has indeed become one of the key translational technologies driving regenerative medicine from the laboratory to the clinic. According to the results of this study, the meniscus bionic scaffold prepared by LDM technology exhibits significant advantages in biomechanical properties, biological activity, and personalized treatment. In the future, through material optimization (such as nanocomposites) and combined with cell therapy (such as stem cell‐seeded scaffolds), it may achieve complete functional regeneration, replacing some meniscus transplantation surgeries.

Even though this study demonstrated great potential in the in situ treatment of meniscus, this developed scaffold is still in its infancy, so there is a long way to go to achieve meniscus regeneration in clinical application. Our future studies will focus on the following issues. First, a drawback of this animal model is that rabbits exhibit higher endogenous healing potential than other large animals and humans, making evaluation of the translational potential difficult. Longer‐term outcomes of this scaffold in a large animal model (sheep) will be examined in our next experiment. Second, In the subsequent research, we will further explore how PCL/COL I affects the migration, proliferation, and differentiation of endogenous stem/progenitor cells. Last but not least, we will evaluate the degradation/corrosion behavior of scaffolds in vivo and in vitro in our future work. Despite these limitations, this study still demonstrates that the PCL‐COL I Scaffold prepared by 3D‐LDM technology has good cytocompatibility and excellent mechanical properties, which can regenerate the meniscus in situ, so as to achieve the effect of treating related sports injury diseases.

## 5 CONCLUSION

In summary, we have developed an integrated meniscus scaffold constructed by low‐temperature deposition 3DP technology, with composition and structure double bionic natural meniscus, aiming to repair meniscus defects. The use of COL I to mimic the material components of the cellular microenvironment of MFCs, maintain the cell phenotype of MFCs, promote their proliferation and differentiation, and whether Col I can recruit surrounding stem cells and differentiate them into fibrocartilage stem cells needs to be verified by further experiments. On the other hand, PCL has excellent physical properties and solves the problem of insufficient mechanical properties of pure type I collagen scaffolds. In vitro cell experiments also demonstrated that PCL/COL I had good biocompatibility and mechanical properties. Animal model studies have also shown that PCL/COL I scaffolds can effectively repair meniscus defects in rabbit knee joints, promote meniscus regeneration, and accelerate meniscus maturation. The above studies showed that the scaffold had a good application prospect in the field of meniscus tissue engineering and provided a theoretical basis for subsequent experiments.

## AUTHOR CONTRIBUTIONS

**Shi Shen and Yujie Li**: Conceptualization, methodology, software, and validation; **Mingxue Chen and Weimin Guo**: Reviewing and editing, visualization, and investigation; **Shuang Gao and Zengzeng Zhang**: Data curation; **Naiqiang Zhuo**: Writing—original draft preparation; **Shuyun Liu and Quanyi Guo**: Writing and reviewing. All the authors contributed to and approved the final version of the manuscript. All authors read and approved the final manuscript.

## FUNDING INFORMATION

This study was funded by grants from the National Key R&D Program of China (2023YFB4605800), the Natural Science Foundation of China (82272481), the Natural Science Foundation of Sichuan Province (2024NSFSC0677), Supported by The Science and Technology Strategic Cooperation Programs of Luzhou Municipal People's Government and Southwest Medical University (2020LZXNYDJ06) and the Southwest Medical University university‐level project (2019ZQN094).

## CONFLICT OF INTEREST STATEMENT

The authors declare that they have no known competing financial interests or personal relationships that could have appeared to influence the work reported in this paper.

## Data Availability

The data that support the findings of this study are available from the corresponding author upon reasonable request.

## References

[1] Walker PS , Erkman MJ . The role of the menisci in force transmission across the knee. Clin Orthop Relat Res. 1975;109(109):184‐192. doi:10.1097/00003086-197506000-00027 1173360

[2] Bansal S , Floyd ER , Kowalski MA , et al. Meniscal repair: the current state and recent advances in augmentation. J Orthop Res. 2021;39(7):1368‐1382. 33751642 10.1002/jor.25021 PMC8249336

[3] Orellana F , Grassi A , Hlushchuk R , et al. Revealing the complexity of meniscus microvasculature through 3D visualization and analysis. Sci Rep. 2024;14(1):10875. 38740845 10.1038/s41598-024-61497-2 PMC11091062

[4] Scott GA , Jolly BL , Henning CE . Combined posterior incision and arthroscopic intra‐articular repair of the meniscus. An examination of factors affecting healing. J Bone Joint Surg Am. 1986;68(6):847‐861. 3755440

[5] Harput G , Guney‐Deniz H , Nyland J , Kocabey Y . Postoperative rehabilitation and outcomes following arthroscopic isolated meniscus repairs: A systematic review. Phys Ther Sport. 2020;45:76‐85. 32688294 10.1016/j.ptsp.2020.06.011

[6] Kurzweil PR , Cannon WD , DeHaven KE . Meniscus repair and replacement. Sports Med Arthrosc Rev. 2018;26(4):160‐164. doi:10.1097/JSA.0000000000000224 30395058

[7] Beaufils P , Becker R , Kopf S , et al. Surgical management of degenerative meniscus lesions: the 2016 ESSKA meniscus consensus. Knee Surg Sports Traumatol Arthrosc. 2017;25(2):335‐346. 28210788 10.1007/s00167-016-4407-4 PMC5331096

[8] Bhan K . Meniscal tears: current understanding, diagnosis, and management. Cureus. 2020;12(6):e8590. 32676231 10.7759/cureus.8590 PMC7359983

[9] Yan W , Maimaitimin M , Wu Y , et al. Meniscal fibrocartilage regeneration inspired by meniscal maturational and regenerative process. Sci Adv. 2023;9(45):eadg8138. doi:10.1126/sciadv.adg8138 37939174 PMC10631723

[10] Ozeki N , Koga H , Sekiya I . Degenerative meniscus in knee osteoarthritis: from pathology to treatment. Life. 2022;12(4):603. 35455094 10.3390/life12040603 PMC9032096

[11] Hohmann E . Treatment of degenerative meniscus tears. Art Ther. 2023;39(4):911‐912. 10.1016/j.arthro.2022.12.002 36872031

[12] Shimomura K , Hamamoto S , Hart DA , Yoshikawa H , Nakamura N . Meniscal repair and regeneration: current strategies and future perspectives. J Clin Orthop Trauma. 2018;9(3):247‐253. [published correction appears in J Clin Orthop Trauma. 2020 Nov‐Dec;11(6):1169‐1171, doi: 10.1016/j.jcot.2020.09.032] 30202157 PMC6128795

[13] Ding G , Du J , Hu X , Ao Y . Mesenchymal stem cells from different sources in meniscus repair and regeneration. Front Bioeng Biotechnol. 2022;10:796367. 35573249 10.3389/fbioe.2022.796367 PMC9091333

[14] Li X , Li D , Li J , et al. Preclinical studies and clinical trials on cell‐based treatments for meniscus regeneration. Tissue Eng Part B Rev. 2023;29(6):634‐670. 37212339 10.1089/ten.TEB.2023.0050

[15] Li H , Li P , Yang Z , et al. Meniscal regenerative scaffolds based on biopolymers and polymers: recent status and applications. Front Cell Dev Biol. 2021;9:661802. 34327197 10.3389/fcell.2021.661802 PMC8313827

[16] Vasiliadis AV , Koukoulias N , Katakalos K . Three‐dimensional‐printed scaffolds for meniscus tissue engineering: opportunity for the future in the Orthopaedic world. J Funct Biomater. 2021;12(4):69. 34940548 10.3390/jfb12040069 PMC8708065

[17] Fitzgerald R , Bass LM , Goldberg DJ , Graivier MH , Lorenc ZP . Physiochemical characteristics of poly‐L‐lactic acid (PLLA). Aesthet Surg J. 2018;38(suppl_1):S13‐S17. doi:10.1093/asj/sjy012 29897517

[18] Kwon H , Brown WE , Lee CA , et al. Surgical and tissue engineering strategies for articular cartilage and meniscus repair. Nat Rev Rheumatol. 2019;15(9):550‐570. 31296933 10.1038/s41584-019-0255-1 PMC7192556

[19] Fu L , Guo W , Guo Q . Editorial: biomaterial advances in cartilage and meniscus regeneration. Front Bioeng Biotechnol. 2022;10:1054268. 36324884 10.3389/fbioe.2022.1054268 PMC9619108

[20] Coluccino L , Gottardi R , Ayadi F , Athanassiou A , Tuan RS , Ceseracciu L . Porous poly(vinyl alcohol)‐based hydrogel for knee meniscus functional repair. ACS Biomater Sci Eng. 2018;4(5):1518‐1527. 33445309 10.1021/acsbiomaterials.7b00879

[21] Zhang Z , Guo W , Gao S , et al. Native tissue‐based strategies for meniscus repair and regeneration. Cell Tissue Res. 2018;373(2):337‐350. 29397425 10.1007/s00441-017-2778-6

[22] Wubneh A , Tsekoura EK , Ayranci C , Uludağ H . Current state of fabrication technologies and materials for bone tissue engineering. Acta Biomater. 2018;80:1‐30. 30248515 10.1016/j.actbio.2018.09.031

[23] Siddiqui N , Asawa S , Birru B , Baadhe R , Rao S . PCL‐based composite scaffold matrices for tissue engineering applications. Mol Biotechnol. 2018;60(7):506‐532. 29761314 10.1007/s12033-018-0084-5

[24] Zhang ZZ , Wang SJ , Zhang JY , et al. 3D‐printed poly(ε‐caprolactone) scaffold augmented with mesenchymal stem cells for Total meniscal substitution: A 12‐ and 24‐week animal study in a rabbit model. Am J Sports Med. 2017;45(7):1497‐1511. 28278383 10.1177/0363546517691513

[25] Bahcecioglu G , Hasirci N , Bilgen B , Hasirci V . A 3D printed PCL/hydrogel construct with zone‐specific biochemical composition mimicking that of the meniscus. Biofabrication. 2019;11(2):025002. 30530944 10.1088/1758-5090/aaf707

[26] Ma H , Xie B , Chen H , et al. Structurally sophisticated 3D‐printed PCL‐fibrin hydrogel meniscal scaffold promotes in situ regeneration in the rabbit knee meniscus. Mater Today Bio. 2024;30:101391. 10.1016/j.mtbio.2024.101391 PMC11715118 39790487

[27] Bahcecioglu G , Bilgen B , Hasirci N , Hasirci V . Anatomical meniscus construct with zone specific biochemical composition and structural organization. Biomaterials. 2019;218:119361. 31336280 10.1016/j.biomaterials.2019.119361

[28] Bahcecioglu G , Hasirci N , Bilgen B , Hasirci V . Hydrogels of agarose, and methacrylated gelatin and hyaluronic acid are more supportive for in vitro meniscus regeneration than three dimensional printed polycaprolactone scaffolds. Int J Biol Macromol. 2019;122:1152‐1162. 30218727 10.1016/j.ijbiomac.2018.09.065

[29] Zhang T , Zhang H , Zhang L , et al. Biomimetic design and fabrication of multilayered osteochondral scaffolds by low‐temperature deposition manufacturing and thermal‐induced phase‐separation techniques. Biofabrication. 2017;9(2):025021. 28462906 10.1088/1758-5090/aa7078

[30] Deponti D , Giancamillo AD , Scotti C , et al. Animal models for meniscus repair and regeneration. J Tissue Eng Regen Med. 2015;9(5):512‐527. 23712959 10.1002/term.1760

[31] Herrera Millar VR , Canciani B , Mangiavini L , et al. Endostatin in 3D fibrin hydrogel scaffolds promotes chondrogenic differentiation in swine neonatal meniscal cells. Biomedicine. 2022;10(10):2415. 10.3390/biomedicines10102415 PMC9598439 36289678

[32] Voskuilen R , Boonen B , Tilman P , Schotanus M , Most J . Demographics are no clinically relevant predictors of patient‐reported knee osteoarthritis symptoms—comprehensive multivariate analysis. J Orthop. 2022;35:85‐92. 36420352 10.1016/j.jor.2022.11.002 PMC9676430

[33] Gloria A , de Santis R , Ambrosio L . Polymer‐based composite scaffolds for tissue engineering. J Appl Biomater Biomech. 2010;8(2):57‐67. 20740467

[34] Tong JB , Sanjiv R , Elderdery A , et al. Current advances in the development of meniscus tissue engineering: narrative review. Med J Malaysia. 2023;78(4):534‐540. 37518929

[35] Guo W , Xu W , Wang Z , et al. Cell‐free strategies for repair and regeneration of meniscus injuries through the recruitment of endogenous stem/progenitor cells. Stem Cells Int. 2018;2018:5310471. 30123286 10.1155/2018/5310471 PMC6079391

[36] Yu Z , Lili J , Tiezheng Z , et al. Development of decellularized meniscus extracellular matrix and gelatin/chitosan scaffolds for meniscus tissue engineering. Biomed Mater Eng. 2019;30(2):125‐132. 30741661 10.3233/BME-191038

[37] Vrancken AC , Buma P , van Tienen TG . Synthetic meniscus replacement: a review. Int Orthop. 2013;37(2):291‐299. doi:10.1007/s00264-012-1682-7 23100123 PMC3560902

[38] Longo UG , Rizzello G , Berton A , et al. A review of preclinical and clinical studies using synthetic materials for meniscus replacement. Curr Stem Cell Res Ther. 2013;8(6):438‐443. doi:10.2174/1574888X1130800061 24016324

[39] Makris EA , Hadidi P , Athanasiou KA . The knee meniscus: structure‐function, pathophysiology, current repair techniques, and prospects for regeneration. Biomaterials. 2011;32(30):7411‐7431. doi:10.1016/j.biomaterials.2011.06.037 21764438 PMC3161498

[40] Twomey‐Kozak J , Jayasuriya CT . Meniscus repair and regeneration: A systematic review from a basic and translational science perspective. Clin Sports Med. 2020;39(1):125‐163. 31767102 10.1016/j.csm.2019.08.003 PMC6884076

[41] Vadodaria K , Kulkarni A , Santhini E , Vasudevan P . Materials and structures used in meniscus repair and regeneration: a review. Biomedicine. 2019;9(1):2. doi:10.1051/bmdcn/2019090102 30794149 PMC6385612

[42] Li Z , Wu N , Cheng J , et al. Biomechanically, structurally and functionally meticulously tailored polycaprolactone/silk fibroin scaffold for meniscus regeneration. Theranostics. 2020;10(11):5090‐5106. 32308770 10.7150/thno.44270 PMC7163455

[43] Bilgen B , Jayasuriya CT , Owens BD . Current concepts in meniscus tissue engineering and repair. Adv Healthc Mater. 2018;7(11):e1701407. 29542287 10.1002/adhm.201701407 PMC6176857

[44] Gao S , Guo W , Chen M , et al. Fabrication and characterization of electrospun nanofibers composed of decellularized meniscus extracellular matrix and polycaprolactone for meniscus tissue engineering. J Mater Chem B. 2017;5(12):2273‐2285. 32263618 10.1039/c6tb03299k

[45] Li H , Liao Z , Yang Z , et al. 3D printed poly(ε‐caprolactone)/meniscus extracellular matrix composite scaffold functionalized with Kartogenin‐releasing PLGA microspheres for meniscus tissue engineering. Front Bioeng Biotechnol. 2021;9:662381. 33996783 10.3389/fbioe.2021.662381 PMC8119888

[46] González‐Duque MI , Flórez AM , Torres MA , Fontanilla MR . Composite zonal scaffolds of collagen I/II for meniscus regeneration. ACS Biomater Sci Eng. 2024;10(4):2426‐2441. 38549452 10.1021/acsbiomaterials.3c01737

[47] Roncada T , Blunn G , Roldo M . Collagen and alginate hydrogels support chondrocytes Redifferentiation in vitro without supplementation of exogenous growth factors. ACS Omega. 2024;9(19):21388‐21400. 38764657 10.1021/acsomega.4c01675 PMC11097186

[48] Guo W , Chen M , Wang Z , et al. 3D‐printed cell‐free PCL‐MECM scaffold with biomimetic micro‐structure and micro‐environment to enhance in situ meniscus regeneration. Bioact Mater. 2021;6(10):3620‐3633. 33869902 10.1016/j.bioactmat.2021.02.019 PMC8039774

[49] Savin G , Caillol S , Bethry A , et al. Collagen/polyester‐polyurethane porous scaffolds for use in meniscal repair. Biomater Sci. 2024;12(11):2960‐2977. 38682257 10.1039/d4bm00234b

[50] Berni M , Marchiori G , Cassiolas G , et al. Anisotropy and inhomogeneity of permeability and fibrous network response in the pars intermedia of the human lateral meniscus. Acta Biomater. 2021;135:393‐402. 34411754 10.1016/j.actbio.2021.08.020

[51] van Bochove B , Hannink G , Buma P , Grijpma DW . Preparation of designed poly(trimethylene carbonate) meniscus implants by stereolithography: challenges in stereolithography. Macromol Biosci. 2016;16(12):1853‐1863. 27748548 10.1002/mabi.201600290

[52] Zhang ZZ , Jiang D , Ding JX , et al. Role of scaffold mean pore size in meniscus regeneration. Acta Biomater. 2016;43:314‐326. 27481291 10.1016/j.actbio.2016.07.050

[53] Yang Y et al. Biomimetic anisotropic reinforcement architectures by electrically assisted nanocomposite 3D printing. Adv Mater. 2017;29(11). 10.1002/adma.201605750 PMC7032659 28185341

[54] Han D , Wang W , Gong J , Ma Y , Li Y . Collagen‐hydroxyapatite based scaffolds for bone trauma and regeneration: recent trends and future perspectives. Nanomedicine. 2024;19(18–20):1689‐1709. 39163266 10.1080/17435889.2024.2375958 PMC11389751

[55] Song T , Zhou J , Shi M , et al. Osteon‐mimetic 3D nanofibrous scaffold enhances stem cell proliferation and osteogenic differentiation for bone regeneration. Biomater Sci. 2022;10(4):1090‐1103. 35040827 10.1039/d1bm01489g

[56] Alavi MS , Memarpour S , Pazhohan‐Nezhad H , et al. Applications of poly(lactic acid) in bone tissue engineering: A review article. Artif Organs. 2023;47(9):1423‐1430. 37475653 10.1111/aor.14612

[57] Hu G , Bao L , Li G , Chen L , Hong FF . Vascular cells responses to controlled surface structure and properties of bacterial nanocellulose artificial blood vessel after mercerization. Carbohydr Polym. 2023;306:120572. 36746593 10.1016/j.carbpol.2023.120572

[58] Ebrahimi Z , Irani S , Ardeshirylajimi A , Seyedjafari E . Enhanced osteogenic differentiation of stem cells by 3D printed PCL scaffolds coated with collagen and hydroxyapatite. Sci Rep. 2022;12(1):12359. 35859093 10.1038/s41598-022-15602-y PMC9300684

[59] Arango‐Santander S . Bioinspired topographic surface modification of biomaterials. Materials. 2022;15(7):2383. 35407716 10.3390/ma15072383 PMC8999667

[60] Du MZ , Dou Y , Ai LY , et al. Meniscus heterogeneity and 3D‐printed strategies for engineering anisotropic meniscus. Int J Bioprint. 2023;9(3):693. 37273997 10.18063/ijb.693 PMC10236485

